# Association between human papillomavirus infection or immunization and risk for rheumatoid arthritis

**DOI:** 10.3389/fimmu.2023.1130217

**Published:** 2023-04-14

**Authors:** Guangxia Yang, Zeqin Ren, Kai Wang

**Affiliations:** ^1^ Department of Rheumatology, Affiliated Hospital of Jiangnan University, Wuxi, Jiangsu, China; ^2^ Department of Rehabilitation, The First Affiliated Hospital of Dali University, Dali, Yunnan, China; ^3^ Department of Rheumatology, The Affiliated Huaian No.1 People’s Hospital of Nanjing Medical University, Huai’an, Jiangsu, China

**Keywords:** human papillomavirus (HPV), rheumatoid arthritis, infection, immunization (vaccination), autoimmune disease

## Abstract

**Background:**

Human papillomavirus (HPV) is a virus primarily transmitted through sexual contact. Little is known about the association between HPV infection or immunization and the risk for rheumatoid arthritis (RA). The purpose of this study was to evaluate whether HPV infection or immunization is related to the risk for RA in adults.

**Methods:**

Data were obtained from the 2007-2016 National Health and Nutrition Examination Survey (NHANES). We developed three independent multivariate logistic regression models to evaluate the association between HPV infection or immunization and the risk for RA in adults.

**Results:**

Finally, we analyzed 15,677 and 8,944 subjects, respectively. In all models, HPV infection was positively associated with an increased prevalence of RA in adults aged 18-59 years, with the highest value of the odds ratio (OR) in model 2 (after weighting: OR 1.095, 95% CI 1.092, 1.097), whereas HPV immunization significantly reduced the prevalence of RA in adults aged 18-59 years, with the lowest OR in model C (after weighting: OR 0.477, 95% CI 0.472, 0.481). These associations persisted after correction for confounders such as age, sex, race, education level, marital status, smoking, diabetes, hypertension, hyperlipidemia, and BMI.

**Conclusion:**

In summary, our study suggests that HPV infection is positively associated with the prevalence of RA in adults, and HPV immunization can reduce the prevalence of RA in adults. However, our findings need more powerful to prove these associations through rigorously designed prospective studies.

## Introduction

Rheumatoid arthritis (RA) is a chronic, progressive, systemic autoimmune disease characterized by synovitis. The prevalence of RA varies globally, with an age-standardized prevalence of 246.6 per 100,000, and has increased in recent years (1, 2). The etiology of RA has not yet been fully elucidated and may be related to a variety of genetic and environmental factors, of which the strongest risk factors include female gender, family history of RA, genetic factors, and exposure to tobacco smoke (3). In addition, infections are also considered to be an important cause of RA, and some bacterial and viral infections including periodontal bacteria, Epstein-Barr virus, parvovirus B19 and Chikungunya virus are considered to be associated with RA (4–7). The mechanism of how infection affects RA remains undetermined, and it is commonly believed that the antigen of the pathogen may trigger cross-reactivity between autoantigen and viral proteins through a molecular mimicry mechanism (7).

A population-based cohort study showed that the adjusted hazard ratio of human papillomavirus (HPV) infection to RA was 1.40, suggesting that HPV infection may be a predisposing factor for RA (8). The mechanism of how HPV infection affects RA has not been determined, but it may be related to HPV-47 E345-362 antigen peptide, which has homology with the epitope of RA autoantigen polyfilin. Patients with RA have high titers of autoantibodies against the virus citrullinated antigen peptide, suggesting that HPV infection and its antigen may play a role in the pathogenesis of RA (9). The HPV vaccine is an effective public health measure to control HPV infection and subsequent complications. HPV vaccine can not only significantly reduce the infection of young people but also reduce the infection of unvaccinated people (10, 11). However, there is a lack of research on whether HPV vaccination reduces the risk for RA. In this population-based cohort study, we aimed to explore the relationship between HPV infection or immunization and the prevalence of RA in adults.

## Method

### Study design and data sources

Data used in this study were obtained from five survey cycles (2007-2008, 2009-2010, 2011-2012, 2013-2014, 2015-2016) of the National Health and Nutrition Examination Survey (NHANES). NHANES is a cross-sectional study conducted by the National Center for Health Statistics (NCHS) to assess the nutritional and health status of the non-institutional American civilian population. The survey is conducted every 2 years and collected information including demographic data, dietary data, examine data, laboratory data and questionnaire data. Details of participants’ demographics, socioeconomic, dietary factors, and medical and health conditions were collected through family interviews. Standardized medical and physical examinations are performed by trained medical personnel through the collection of biological samples such as serum and urine in specially equipped mobile examination centers (MECs). The protocol for the NHANES study was reviewed and approved by the NCHS Study Ethics Review Committee and all participants signed informed consent forms. Given the age range for HPV polymerase chain reaction (PCR) test data was 18-59 years, we included participants aged 18-59 years with complete data on HPV PCR test results, history of HPV vaccination and history of RA in our analysis.

### Measurements of HPV infection

HPV infection was assessed by laboratory data. Samples included vaginal swabs and oral rinses. Participants with “positive” test results were classified as HPV infected group, those with “negative” test results were classified as HPV uninfected group, and those participants with “Inadequate” test results were excluded. Detailed procedures and methods can be found in the laboratory procedures manual.

### Measurements of HPV immunization

HPV immunization was assessed through a self-report questionnaire vaccination question. Participants who answered “yes” to the question on HPV vaccination were classified as the HPV vaccinated group, and those who answered “no” were classified as the HPV unvaccinated group, excluding those who answered “refused” and “don’t know”. Missing data were also excluded.

### Measurements of RA

The assessment of RA was determined by self-report questionnaire questions. These questions included “Has a doctor or other health professional ever told you that you had arthritis?” and “Which type of arthritis was it?”. Participants who answered “RA” to the latter question were classified in the RA group, while those who answered “no” to the former question and “osteoarthritis, degenerative arthritis, psoriasis, and others” to the latter question were classified in the non-RA group.

### Covariates

We included confounding factors that might influence the relationship between proposed HPV infection or immunization and RA as covariates, based on other published studies (12, 13). The covariates included age, sex, race, education level, marital status, smoking status, diabetes, hypertension, hyperlipidemia, and BMI. We analyzed the proportion of missing values in the covariates using the R package mice with multiple imputation of the missing values (14).

### Statistical analysis

Distribution characteristics were expressed as standard deviation (SD) for continuous variables and as the number of cases (n) and percentage (%) for categorical variables. The chi-square test was used to assess differences in categorical variables, and the Mann-Whitney test was used to assess differences in continuous variables. Risk ratio (RR) was used to evaluate the association between HPV infection or immunization with the risk for RA among all participants. Subsequently, we used the R package Matching for propensity score matching (1:1) to match HPV infection or HPV immunization corresponding controls to reduce heterogeneity and selection bias (15). Standardized mean difference was used to assess the effect of matching. We constructed three multivariate logistic regression (MLR) models separately to estimate the odds ratio (OR) and corresponding confidence intervals (CI) for the risk of RA associated with HPV infection or HPV immunization. Model 1 and model A were adjusted for age and sex only. Model 2 and model B were adjusted for confounders with a missing proportion of values below 5% (16). Model 3 and model C were further adjusted for all confounders. Because NHANES uses a complex stratified multistage sampling design, weighted analyses were performed once every 10 years in order to balance patient characteristics between groups. All statistical analyses were performed using R software (version 4.2.2), and P values less than 0.05 were considered statistically significant.

## Results

### Baseline characteristics of study participants on HPV infection

**Figure 1:**
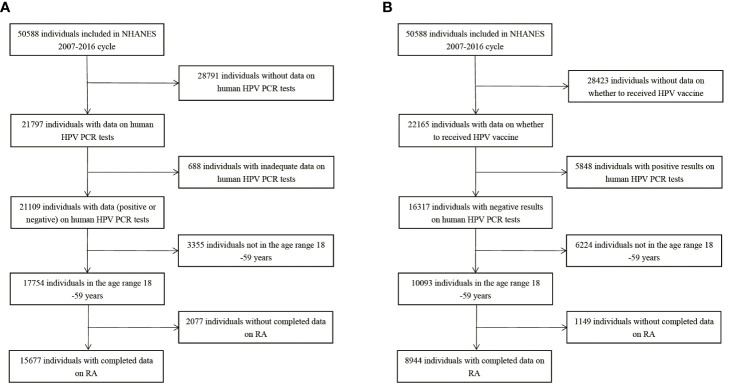
Flow chart of population included in our final analysis, NHANES 2007-2016. **(A)** Flow chart of HPV infection cohort. **(B)** Flow chart of HPV immunization cohort. HPV, Human papillomavirus; PCR, polymerase chain reaction; RA, rheumatoid arthritis.

**Figure 2:**
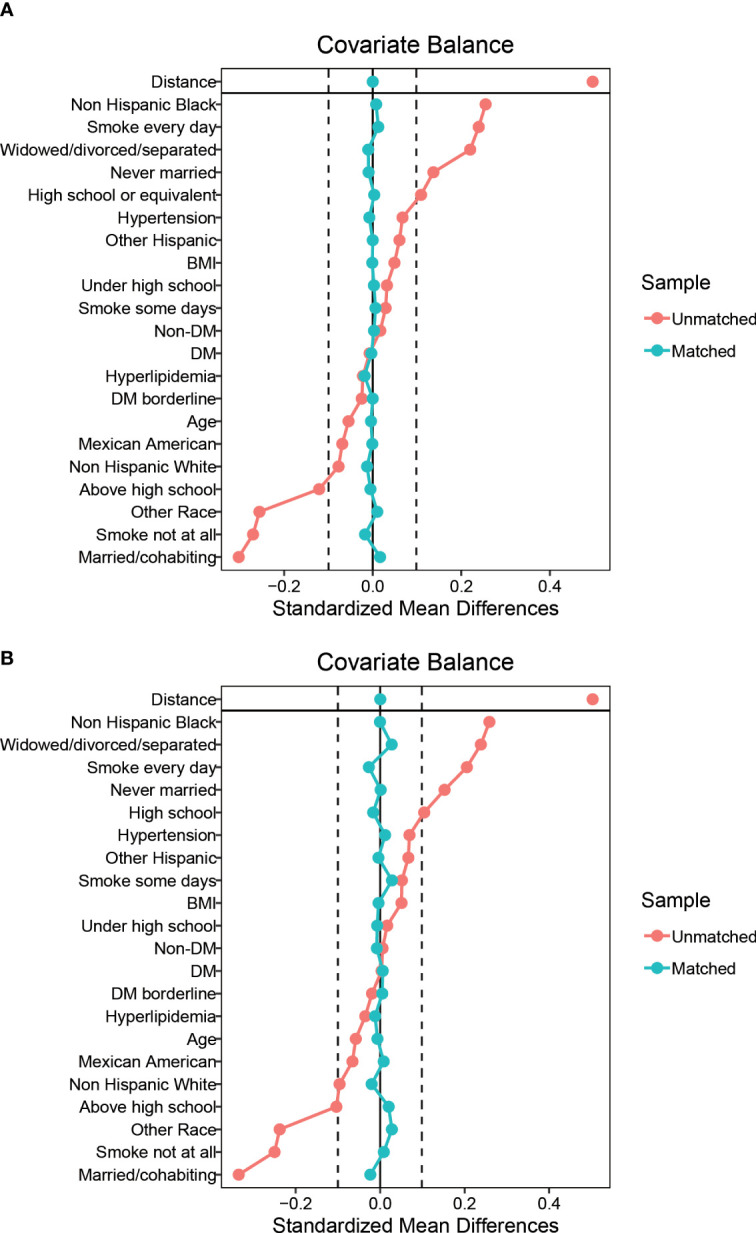
Balance diagnostics plot for HPV infection data, before and after matching. **(A)** Standardized differences shown for each baseline confounder variable of HPV infection cohort before weighting. **(B)** Standardized differences shown for each confounder variable of HPV infection cohort after weighting. Vertical dotted lines indicate the desired balance level. The X-axis represents the standardized differences value, and the Y-axis represents baseline variables. HPV, Human papillomavirus; BMI, body mass index; DM, diabetes mellitus.

15,677 participants were included in the study of HPV infection and RA (**Figure 1A**), and 8,944 participants were included in the study of HPV immunity and RA (**Figure 1B**). **Table 1** and **Supplementary Table 1** summarized the unweighted and weighted baseline characteristics of HPV infected and uninfected groups in the HPV infection cohort and showed similar distributions for the HPV infected and HPV uninfected groups due to good balanced matching (**Figure 2**). **Table 2** summarized the unweighted and weighted baseline characteristics of RA and non-RA groups in HPV infection cohort. There were three covariates whose missing ratio is greater than 5%, namely marital status, hyperlipidemia and smoking status (**Supplementary Figure 1**). The average age of all participants included was 38.66 ± 11.35 years (after weighting), with non-Hispanic whites accounting for the largest proportion, followed by non-Hispanic blacks, and other races the least. Most of the participants have higher education level than senior high school, and their marriages are in good condition. Notably, before weighting, the RA group had a significantly higher proportion of non-Hispanic whites and non-Hispanic blacks and a significantly lower proportion of other races than the non-RA group. Compared to the RA group, participants in the non-RA group had better education levels, lower BMI, and smoked relatively less (**Table 2**).

**Table 1:** Demographic characteristics of HPV infection cohort before weighting.

	Before propensity score matched			After propensity score matched		
	Infected	Uninfected	*P* value	Infected	Uninfected	*P* value
	N = 5668	N = 10009		N = 5202	N = 5202	
Age (years, mean ± SD)	38.37 ± 11.53	39 ± 11.23	**0.001**	38.38 ± 11.50	38.44 ± 11.49	0.793
Gender, n (%)						
Male	1922 (33.9)	4764 (47.6)	**<0.001**	1836 (35.3)	1865 (35.9)	0.553
Female	3746 (66.1)	5245 (52.4)	**<0.001**	3366 (64.7)	3337 (64.1)	0.553
Race/ethnicity, n (%)						
Mexican American	848 (15)	1743 (17.4)	**<0.001**	802 (15.4)	856 (16.5)	0.148
Other Hispanic	685 (12.1)	1013 (10.1)	**<0.001**	615 (11.8)	634 (12.2)	0.567
Non-Hispanic White	1988 (35.1)	3879 (38.8)	**<0.001**	1892 (36.4)	1877 (36.1)	0.76
Non-Hispanic Black	1630 (28.8)	1724 (17.2)	**<0.001**	1381 (26.5)	1329 (25.5)	0.245
Other race	517 (9.1)	1650 (16.5)	**<0.001**	512 (9.8)	506 (9.7)	0.843
Education, n (%)						
Under high school	1265 (22.3)	2100 (21.0)	**0.05**	1167 (22.4)	1172 (22.5)	0.907
High school or equivalent	1396 (24.6)	1995 (19.9)	**<0.001**	1251 (24)	1249 (24)	0.963
Above high school	3007 (53.1)	5914 (59.1)	**<0.001**	2784 (53.5)	2781 (53.5)	0.953
Marital status, n (%)						
Married/cohabiting	2835 (50)	8520 (65.1)	**<0.001**	2717 (52.2)	2792 (53.7)	0.141
Widowed/divorced/separated	1160 (20.5)	1160 (11.6)	**<0.001**	996 (19.1)	923 (17.7)	0.065
Never married	1673 (29.5)	2329 (23.3)	**<0.001**	1489 (28.6)	1487 (28.6)	0.965
BMI (mean ± SD)	29.28 ± 7.34	28.92 ± 7.2	**0.003**	29.22 ± 7.34	29.24 ± 7.43	0.835
Smoking status, n (%)						
Every day	3001 (52.92)	4104 (41)	0.073	2689 (51.7)	2620 (50.4)	0.176
Some days	680 (12)	1106 (11.1)	**<0.001**	612 (11.8)	620 (11.9)	0.808
Not at all	5668 (35.1)	1009 (47.9)	**<0.001**	1901 (36.5)	1962 (37.7)	0.216
Diabetes, n (%)						
Yes	542 (9.6)	978 (9.8)	0.671	503 (9.7)	513 (9.9)	0.741
No	5023 (88.6)	8816 (88.1)	0.313	4600 (88.4)	4590 (88.2)	0.76
Borderline	103 (1.8)	215 (2.1)	0.158	99 (1.9)	99 (1.9)	1.000
Hypertension, n (%)	1363 (24)	2119 (21.2)	**<0.001**	1226 (23.6)	1218 (23.4)	0.853
Hyperlipidemia, n (%)	1389 (24.5)	2548 (25.5)	0.187	1294 (24.9)	1298 (25)	0.928

The bold values means statistical significance.

HPV, Human papillomavirus; BMI, body mass index; DM, diabetes mellitus.

**Table 2:** Demographic characteristics of HPV infection cohort before and after weighting, grouped by RA.

	Before weighting			After weighting		
	RA	Non-RA	*P* value	RA	Non-RA	*P* value
	N = 533	N = 15144		N = 4590282	N = 125591166	
Age (years, mean ± SD)	47.52 ± 8.81	38.46 ± 11.3	**<0.001**	47.09 ± 8.81	38.35 ± 11.31	**<0.001**
Gender, n (%)						
Male	162 (30.4)	6524 (43.1)	**<0.001**	1369484 (29.8)	53538117 (42.6)	**<0.001**
Female	371 (69.6)	8620 (56.9)	**<0.001**	3220798 (70.2)	72053048 (57.4)	**<0.001**
Race/ethnicity, n (%)						
Mexican American	77 (14.4)	2514 (16.6)	0.188	646461 (14.1)	20872893 (16.6)	**<0.001**
Other Hispanic	60 (11.3)	1638 (10.8)	0.748	561032 (12.2)	13729545 (10.9)	**<0.001**
Non-Hispanic White	220 (41.3)	5647 (37.3)	<0.001	1961892 (42.7)	46380636 (36.9)	**<0.001**
Non-Hispanic Black	150 (28.1)	3204 (21.2)	<0.001	1198403 (26.1)	26636620 (21.2)	**<0.001**
Other race	26 (4.9)	2141 (14.1)	<0.001	222495 (4.8)	17971472 (14.3)	**<0.001**
Education, n (%)						
Under high school	159 (29.8)	3206 (21.2)	<0.001	1386620 (30.2)	26371409 (21)	**<0.001**
High school or equivalent	119 (22.3)	3272 (21.6)	0.691	919153 (20)	27258359 (21.7)	**<0.001**
Above high school	255 (47.8)	8666 (57.2)	**<0.001**	2284509 (49.8)	71961397 (57.3)	**<0.001**
Marital status, n (%)						
Married/cohabiting	292 (54.8)	9063 (59.8)	**0.019**	2585260 (56.3)	74594680 (59.4)	**<0.001**
Widowed/divorced/ separated	167 (31.3)	2153 (14.2)	**<0.001**	1374186 (29.9)	18199862 (14.5)	**<0.001**
Never married	74 (13.9)	3928 (25.9)	**<0.001**	630836 (13.7)	32796624 (26.1)	**<0.001**
BMI (mean ± SD)	32.35 ± 8.44	28.93 ± 7.18	**<0.001**	33.12 ± 8.5	29.37 ± 7.33	**<0.001**
Smoking status, n (%)						
Every day	264 (49.5)	6841 (45.2)	**0.047**	2170220 (47.3)	56628783 (45.1)	**<0.001**
Some days	60 (11.3)	1726 (11.4)	0.92	616016 (13.4)	14574251 (11.6)	**<0.001**
Not at all	209 (39.2)	6577 (43.4)	0.053	1804046 (39.3)	54388131 (43.3)	**<0.001**
Diabetes, n (%)						
Yes	37 (6.9)	1483 (9.8)	0.671	282898 (6.2)	11373875 (9.1)	**<0.001**
No	475 (89.1)	13364 (88.2)	0.539	4085260 (89)	111517920 (88.8)	**<0.001**
Borderline	21 (3.9)	297 (2)	**0.001**	222125 (4.8)	2699370 (2.1)	**<0.001**
Hypertension, n (%)	255 (47.8)	3227 (21.3)	**<0.001**	2236459 (48.7)	27047970 (21.5)	**<0.001**
Hyperlipidemia, n (%)	245 (46)	3692 (24.4)	**<0.001**	2156665 (47)	30289529 (24.1)	**<0.001**

The bold values means statistical significance.

HPV, Human papillomavirus; BMI, body mass index; DM, diabetes mellitus.

### Association between HPV infection and RA

**Figure 3:**
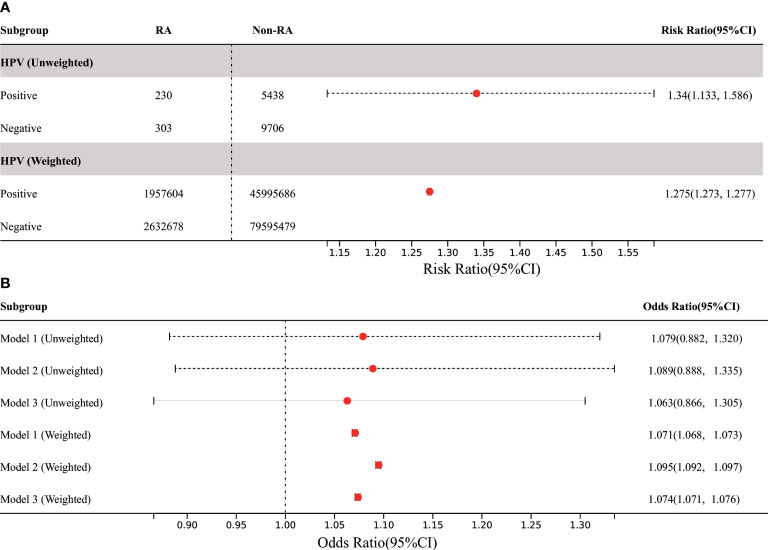
Association between HPV infection and RA in US adults. **(A)** Among all participants, the prevalence of RA in adults was significantly increased in the HPV-infected group compared with the HPV-uninfected control group. **(B)** In the three MLR models, the adjusted OR value of HPV infection was positively correlated with RA, but it was only statistically significant in weighted models. Model 1 was only adjusted for age and gender. Model 2 was adjusted for the confounding covariates with the numerical missing ratio below 5%. Model 3 was further adjusted for all confounding covariates. HPV, Human papillomavirus; BMI, body mass index; DM, diabetes mellitus; MLR: multivariate logistic regression.

In all participants, compared with the control group without HPV infection, the risk of RA in HPV infected group was significantly higher (before weighting: RR 1.34% CI 1.133,1.586; after weighting: RR 1.275, 95% CI 1.273, 1.277), however, this result has not been adjusted for confounding factors (**Figure 3**). In order to further adjust the confounding factors, we conducted MLR analysis after matching. The results showed that in the three models, the adjusted OR values of HPV infection were positively correlated with RA, which was statistically significant after weighting (**Figure 3**). The highest OR was found in model 2 (after weighting: OR 1.095, 95% CI 1.092, 1.097).

### Baseline characteristics of study participants on HPV immunization

**Figure 4:**
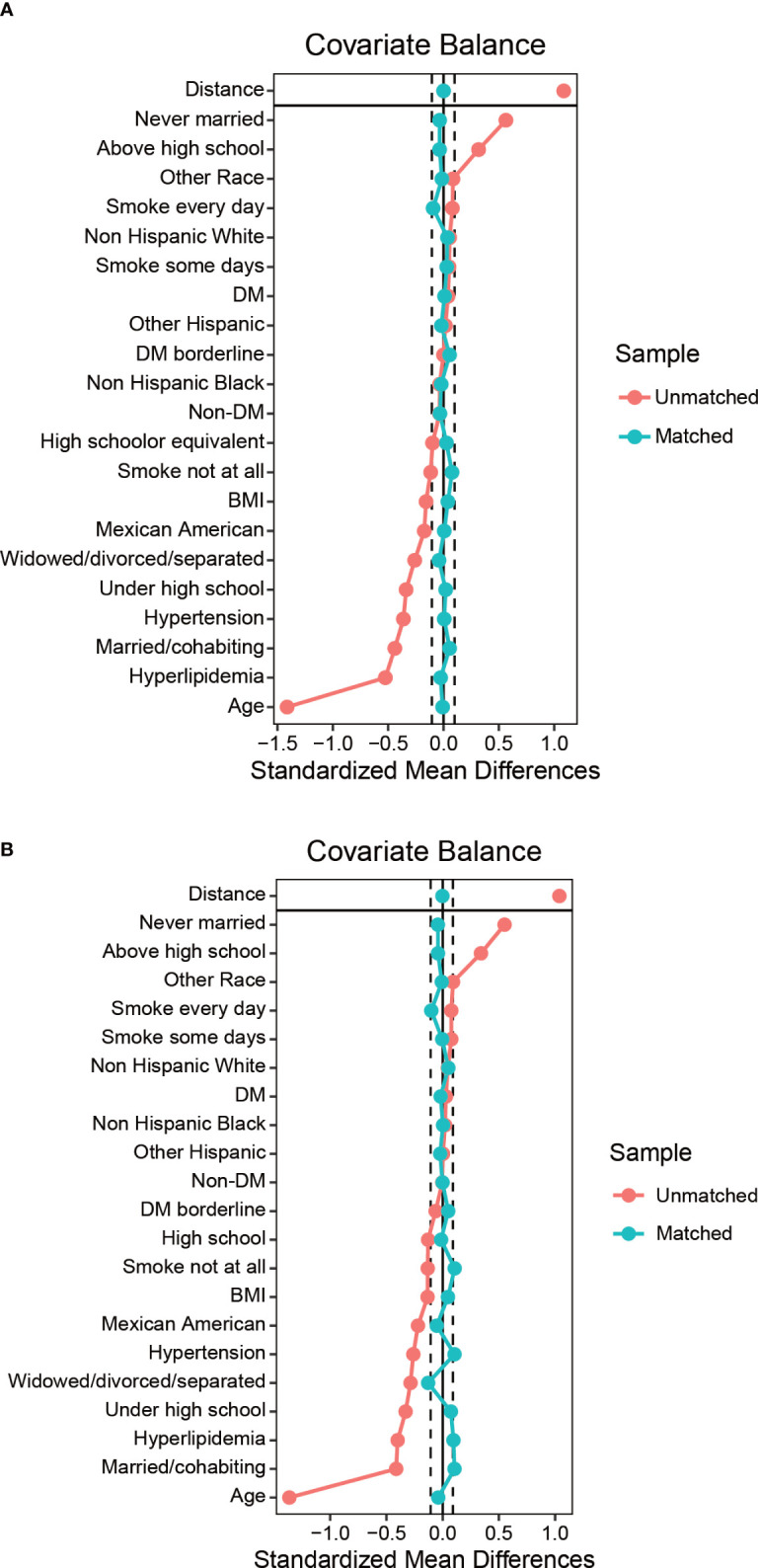
Balance diagnostics plot for HPV immunization data, before and after matching. **(A)** Standardized differences shown for each baseline confounder variable of HPV immunization cohort before weighting. **(B)** Standardized differences shown for each confounder variable of HPV immunization cohort after weighting. Vertical dotted lines indicate the desired balance level. The X-axis represents the standardized differences value, and the Y-axis represents baseline variables. HPV, Human papillomavirus; BMI, body mass index; DM, diabetes mellitus.

**Table 3** and **Supplementary Table 2** summarized the unweighted and weighted baseline characteristics of participants in the HPV vaccinated and HPV unvaccinated groups in the HPV Immunization cohort. After propensity score matching, the two groups also show similar distribution (**Figure 4**). Four covariates had a proportion of missing values greater than 5%, namely marital status, hyperlipidemia, hypertension, and smoking status (**Supplementary Figure 1**). **Table 4** summarized the unweighted and weighted baseline characteristics of participants in the RA and non-RA groups in the HPV immunization cohort. The average age of all included participants was 39.62 ± 11.15 years (after weighting), and the proportions of race, education level, and marital status were similar to those of the HPV infection cohort. Interestingly, comparisons between the RA and non-RA groups also showed similar results to the HPV infection cohort.

**Table 3:** Demographic characteristics of HPV immunization cohort before weighting.

	Before propensity score matched			After propensity score matched		
	Vaccinated	Unvaccinated	*P* value	Vaccinated	Unvaccinated	*P* value
	N = 517	N = 8427		N = 497	N = 497	
Age (years, mean ± SD)	27.96 ± 8.49	39.95 ± 10.95	**<0.001**	28.21 ± 8.56	28.27 ± 8.12	0.909
Gender, n (%)						
Male	113 (21.9)	3276 (38.9)	**<0.001**	113 (22.7)	116 (23.3)	0.821
Female	404 (78.1)	5151 (61.1)	**<0.001**	384 (77.3)	381 (76.7)	0.821
Race/ethnicity, n (%)						
Mexican American	57 (11)	1388 (16.5)	**0.001**	57 (11.5)	59 (11.9)	0.843
Other Hispanic	55 (10.6)	853 (10.1)	0.706	51 (10.3)	62 (12.5)	0.272
Non-Hispanic White	209 (40.4)	3178 (37.7)	0.217	200 (40.2)	193 (38.8)	0.65
Non-Hispanic Black	85 (16.4)	1494 (17.7)	0.456	83 (16.7)	83 (16.7)	1.000
Other race	111 (21.5)	1514 (18)	0.045	106 (21.3)	100 (20.1)	0.639
Education level, n (%)						
Under high school	54 (10.4)	1751 (20.8)	**0.05**	54 (10.9)	66 13.3)	0.242
High school or equivalent	82 (15.9)	1641 (19.5)	**0.043**	81 (16.3)	78 (15.7)	0.795
Above high school	381 (73.7)	5035 (59.7)	**<0.001**	362 (72.8)	353 (71)	0.525
Marital status, n (%)						
Married/cohabiting	230 (44.5)	5592 (66.4)	**<0.001**	229 (46.1)	212 (42.7)	0.278
Widowed/divorced/ separated	32 (6.2)	1051 (12.5)	**<0.001**	31 (6.2)	39 (7.8)	0.321
Never married	255 (49.3)	1784 (21.2)	**<0.001**	237 (47.7)	246 (49.5)	0.568
BMI (mean ± SD)	27.87 ± 7.67	29.08 ± 7.41	**<0.001**	27.87 ± 7.67	28.07 ± 8.21	0.687
Smoking status, n (%)						
Every day	255 (49.3)	3819 (45.3)	0.076	244 (49.1)	236 (47.5)	0.612
Some days	66 (12.8)	935 (11.1)	0.242	65 (13.1)	59 (11.9)	0.565
Not at all	196 (37.9)	3669 (43.5)	**0.012**	188 (37.8)	202 (40.6)	0.363
Diabetes, n (%)						
Yes	26 (5)	349 (4.1)	0.328	24 (4.8)	25 (5)	0.884
No	485 (93.8)	7978 (94.7)	0.399	467 (94)	462 (93)	0.521
Borderline	6 (1.2)	98 (1.2)	0.996	6 (1.2)	10 (2)	0.313
Hypertension, n (%)	56 (10.8)	1862 (22.1)	**<0.001**	55 (11.1)	58 (11.7)	0.764
Hyperlipidemia, n (%)	51 (9.9)	2152 (25.5)	0.187	50 (10.1)	55 (11.1)	0.606

The bold values means statistical significance.

HPV, Human papillomavirus; BMI, body mass index; DM, diabetes mellitus.

**Table 4:** Demographic characteristics of HPV immunization cohort before and after weighting, grouped by RA.

	Before weighting			After weighting		
	RA	Non-RA	*P* value	RA	Non-RA	*P* value
	N = 281	N = 8663		N = 2268282	N = 66720714	
Age (years, mean ± SD)	47.09 ± 8.5	39 ± 11.16	**<0.001**	47.2 ± 8.57	39.37 ± 11.13	**<0.001**
Gender, n (%)						
Male	70 (24.9)	3319 (38.3)	**<0.001**	490556 (21.6)	25515797 (38.2)	**<0.001**
Female	311 (75.1)	5344 (61.7)	**<0.001**	1777727 (78.4)	41204918 (61.8)	**<0.001**
Race/ethnicity, n (%)						
Mexican American	35 (12.5)	1410 (16.3)	0.087	297604 (13.1)	11874722 (17.8)	**<0.001**
Other Hispanic	41 (14.6)	867 (10)	**0.012**	357612 (15.8)	6347135 (9.5)	**<0.001**
Non-Hispanic White	126 (44.8)	3261 (37.6)	**0.014**	1042612 (46)	24761259 (37.1)	**<0.001**
Non-Hispanic Black	58 (20.6)	1521 (17.6)	0.182	413977 (18.3)	12436207 (18.6)	**<0.001**
Other race	21 (7.5)	1604 (18.5)	**<0.001**	156477 (6.9)	11301391 (16.9)	**<0.001**
Education, n (%)						
Under high school	79 (28.1)	1726 (19.9)	**0.001**	666355 (29.4)	13742359 (20.6)	**<0.001**
High school or equivalent	65 (23.1)	1658 (19.1)	0.095	564309 (24.9)	12890995 (19.3)	**<0.001**
Above high school	137 (48.8)	5279 (60.9)	**<0.001**	1037619 (45.7)	40087361 (60.1)	**<0.001**
Marital status, n (%)						
Married/cohabiting	172 (61.2)	5650 (65.2)	0.165	1433247 (63.2)	43416038 (65.1)	**<0.001**
Widowed/divorced/separated	76 (27)	1007 (11.6)	**<0.001**	583219 (25.7)	8265534 (12.4)	**<0.001**
Never married	33 (11.7)	2006 (23.2)	**<0.001**	251816 (11.1)	15039142 (22.5)	**<0.001**
BMI (mean ± SD)	32.59 ± 8.68	28.89 ± 7.35	**<0.001**	33.01 ± 7.1	29.64 ± 6.58	**<0.001**
Smoking status, n (%)						
Every day	145 (51.6)	3929 (45.4)	**0.038**	1100094 (48.5)	30063106 (45.1)	**<0.001**
Some days	22 (7.8)	979 (11.3)	0.069	203900 (9)	7375291 (11.1)	**<0.001**
Not at all	114 (40.6)	3757 (43.3)	0.363	964289 (42.5)	29256853 (43.8)	**<0.001**
Diabetes, n (%)						
Yes	7 (2.5)	368 (4.2)	0.148	57481 (2.5)	3001784 (4.5)	**<0.001**
No	273 (97.2)	8190 (94.5)	**<0.001**	2193089 (96.7)	62882297 (94.2)	**<0.001**
Borderline	1 (0.4)	103 (1.2)	0.2	17713 (0.8)	828452 (1.2)	**<0.001**
Hypertension, n (%)	128 (45.6)	1790 (20.7)	**<0.001**	1062189 (46.8)	14879638 (22.3)	**<0.001**
Hyperlipidemia, n (%)	123 (43.8)	2080 (24)	**<0.001**	998235 (44)	16617759 (24.9)	**<0.001**

The bold values means statistical significance.

HPV, Human papillomavirus; BMI, body mass index; DM, diabetes mellitus.

### Association between HPV immunization and RA

**Figure 5:**
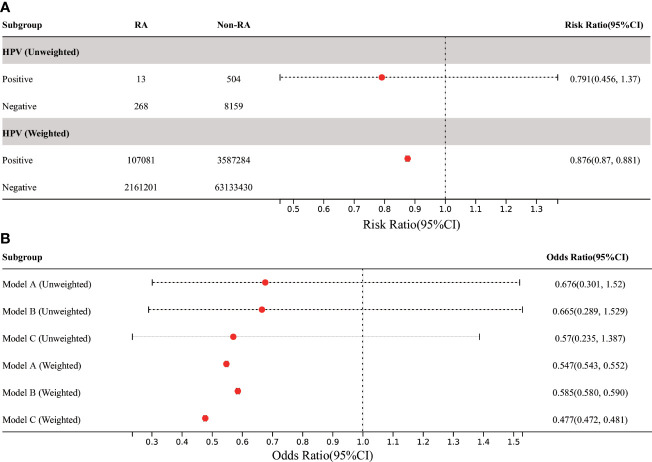
Association between HPV immunization and RA in US adults. **(A)** Among all participants, the prevalence of RA in adults was significantly decreased in the HPV-vaccinated group compared with the HPV-unvaccinated control group. **(B)** In the three MLR models, the adjusted OR value of HPV immunization and RA in American adults aged 18-59 was significantly decreased, but it was only statistically significant in weighted models. Model A was only adjusted for age and gender. Model B was adjusted for the confounding covariates with the numerical missing ratio below 5%. Model C was further adjusted for all confounding covariates. HPV, Human papillomavirus; BMI, body mass index; DM, diabetes mellitus; MLR, multivariate logistic regression.

In all participants, the risk of RA was significantly reduced in the HPV vaccinated group when compared with the HPV unvaccinated group (before weighting: RR 0.791, 95% CI 0.456, 1.37; after weighting: RR was 0.876, 95% CI 0.87, 0.881) (**Figure 5**). Further MLR analysis showed that HPV immunization significantly reduced the prevalence of RA in adults aged 18-59 years in all three models, which was statistically significant after weighting (**Figure 5**). The lowest OR was found in model C (after weighting: OR 0.477, 95% CI 0.472, 0.481).

## Discussion

HPV is a group of small, envelope-free DNA viruses that can infect human skin and mucosal epithelial tissues. It is mainly transmitted mainly through sexual contact and has become a global public health problem. HPV infection can cause a variety of diseases, including cancer, infertility, congenital malformation, and other diseases, the most common of which is cervical cancer (17). The peak of HPV infection is around the age of 20, and its prevalence is inconsistent across reports. In the United States of America (USA), the overall prevalence of oral HPV infection is 11.5% (95% CI: 9.8-13.1%) (18). In China, the prevalence of HPV was 15.0% (95% CI: 14.1-15.9%), with the high-risk genotype and low-risk genotype being 12.1% (95% CI: 11.4-12.7%) and 5.2% (95% CI: 4.8-5.7%) respectively (19). Previous studies have shown that HPV infection may be related to RA, but the correlation has not been fully investigated. Therefore, it is necessary to investigate the relationship between HPV infection or immunization and the risk for RA. Our study is based on the NHANES dataset from 2007 to 2016 and provides new information on the relationship between HPV infection or immunization and the risk for RA. Our research results showed that in the general population, HPV infection is positively correlated with the increased prevalence of RA in adults aged 18-59 years, and these associations persist after adjusting for confounding factors, suggesting that HPV infection may be a predisposing factor for RA.

The HPV vaccine is highly effective in preventing HPV infection, therefore reducing the incidence of cervical cancer. A recent study have shown that HPV immunization programs have significantly reduced the incidence of cervical cancer, especially for women born after September 1, 1995, resulting in almost complete elimination of cervical cancer (20). Consequently, the World Health Organization has urged all countries to introduce the HPV vaccine, with a target coverage of 90%. The HPV vaccine is very effective and well tolerated. Previous studies have demonstrated that HPV vaccination does not increase the risk of autoimmune diseases (21–23). In immunocompromised populations, including patients with RA, incomplete immune response increases the risk of HPV diseases, and the rate of high-grade cervical abnormalities also rises. Therefore, screening should be continued, and HPV vaccination should be actively promoted for these populations (24, 25). Our findings suggest that HPV vaccination significantly reduces the prevalence of RA in adults aged 18-59 years. To our knowledge, this is the first study to explore the relationship between HPV immunization and the risk for RA in a nationally representative sample.

This study is based on the NHANES database and has several limitations. First, the data may be biased due to the exclusion of certain populations, including older adults living in nursing or retirement homes, military personnel living on military bases, prisoners living in federal prisons, the USA citizens living outside the USA, and foreigners living in the USA. Second, only existing data can be collected and analyzed, which may overlook certain important information. Finally, the quality of the data may affect the analysis. NHANES data are self-reported and do not contain comprehensive details such as physician diagnosis of RA, assessment of disease activity, and use of anti-rheumatic drugs. As with all health surveys, the accuracy of the data collected may be influenced by a variety of factors, including respondent honesty, subjective bias, memory, cultural background, social environment, language ability, and education level, making it susceptible to misclassification, nonresponse, and recall bias. Additionally, there are missing values in the data, which may affect the accuracy of the study results. Therefore, more comprehensive studies are needed to further evaluate the relationship between HPV infection or immunization and the risk for RA.

In summary, our study provides new insights into the relationship between HPV infection or immunization and the risk for RA. Our findings indicate that HPV infection is positively associated with RA while HPV immunization reduces the prevalence of RA among adults. This suggests that HPV immunization not only reduce the prevalence of cervical cancer but also significantly reduce the prevalence of RA. Therefore, we recommend that HPV vaccination should be actively promoted, especially for young women, to reduce the risk of developing RA. However, our findings need further validation through rigorously designed prospective studies to provide more robust evidence for this association.

## Data availability statement

Publicly available datasets were analyzed in this study. Data were obtained from the 2007-2016 National Health and Nutrition Examination Survey (NHANES).

## Ethics statement

This study was based on non-identifying data collected from NHANES and did not include any experimental data from human or animal participants; participants were not directly involved in the submission and secondary data were used for analysis. This analysis was considered exempt from Institutional Review Board oversight.

## Author contributions

GY, ZR, and KW contributed to the conception and design of the study. GY and ZR performed data collection. KW performed data analyses. GY and KW wrote the first draft of the manuscript. All authors contributed to the article and approved the submitted version.
