# Supplementary material for: Association between human papillomavirus infection or immunization and risk for rheumatoid arthritis
Source: Front Immunol. 2023 Apr 14;14:1130217. doi: 10.3389/fimmu.2023.1130217 (PMC10146259; doi:10.3389/fimmu.2023.1130217)
Supplement: Supplementary file 1 [file DataSheet_1.docx]

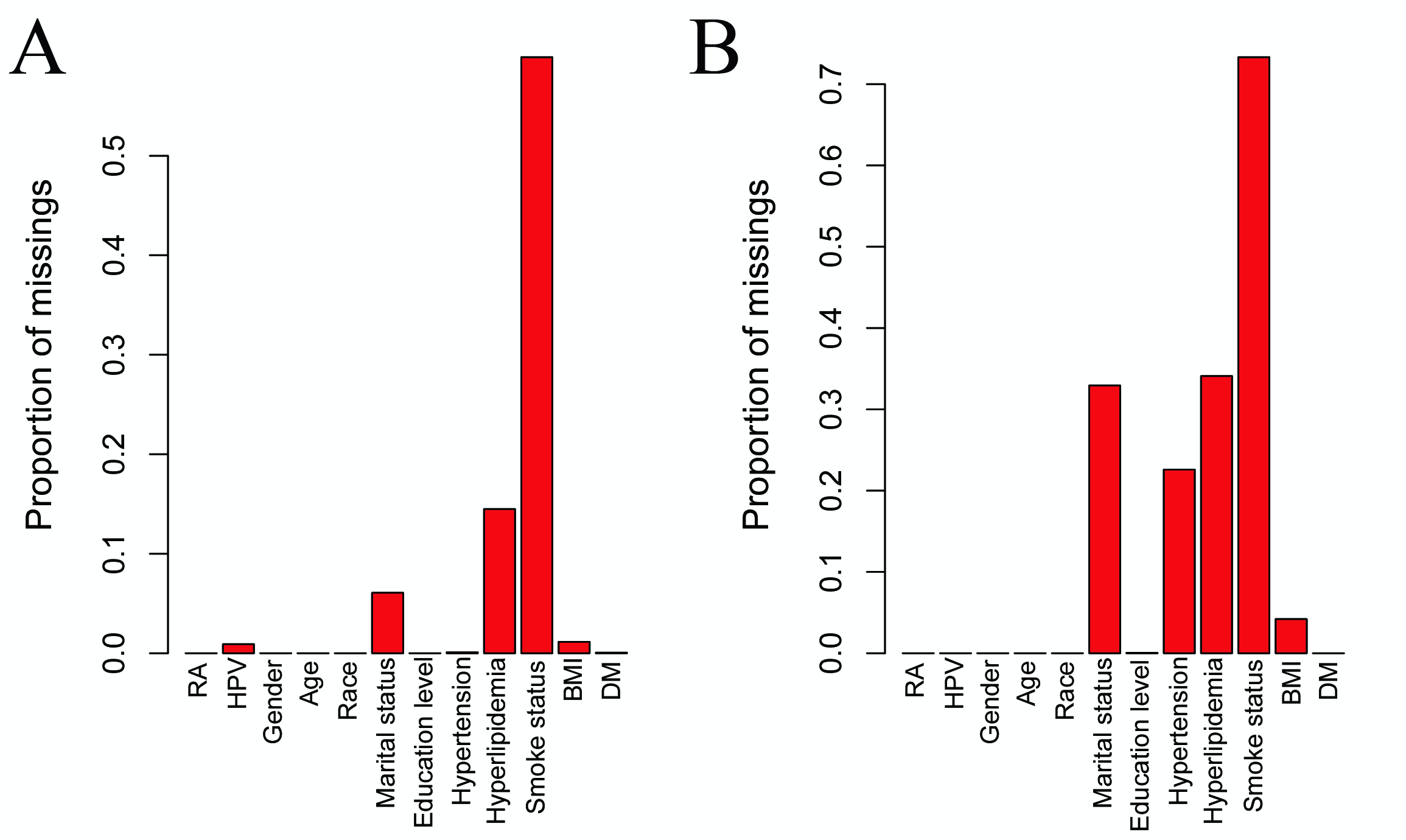


Supplementary Figure 1 Missing ratio of covariates. (A) Missing ratio of covariates of HPV infection cohort. (B) Missing ratio of covariates of HPV immunisation cohort.

HPV, Human papillomavirus; BMI, body mass index; DM, diabetes mellitus.


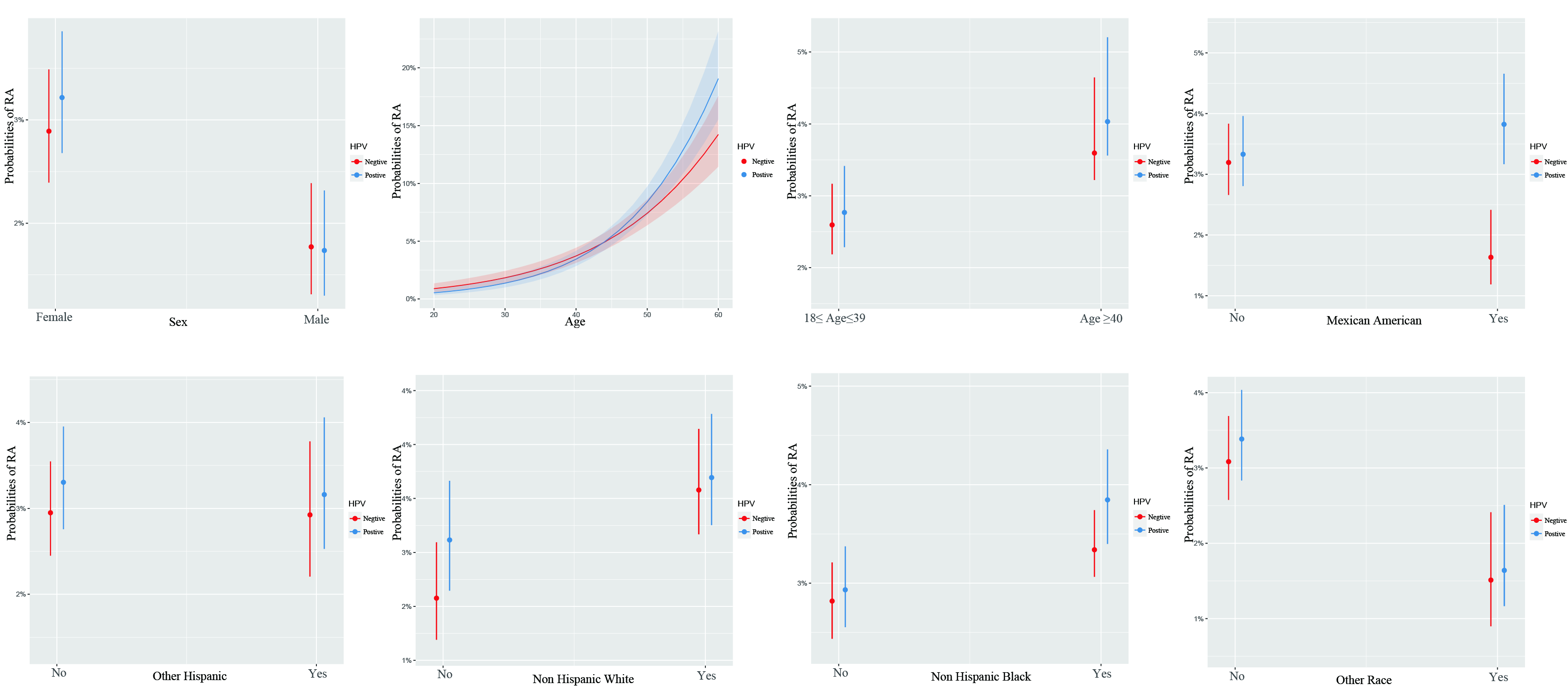


**Supplementary Figure 2** Subgroup analysis of model 2 in HPV infection cohort. Model 2 was adjusted for the confounding covariates with the numerical missing ratio below 5%.

HPV, Human papillomavirus; RA, rheumatoid arthritis.


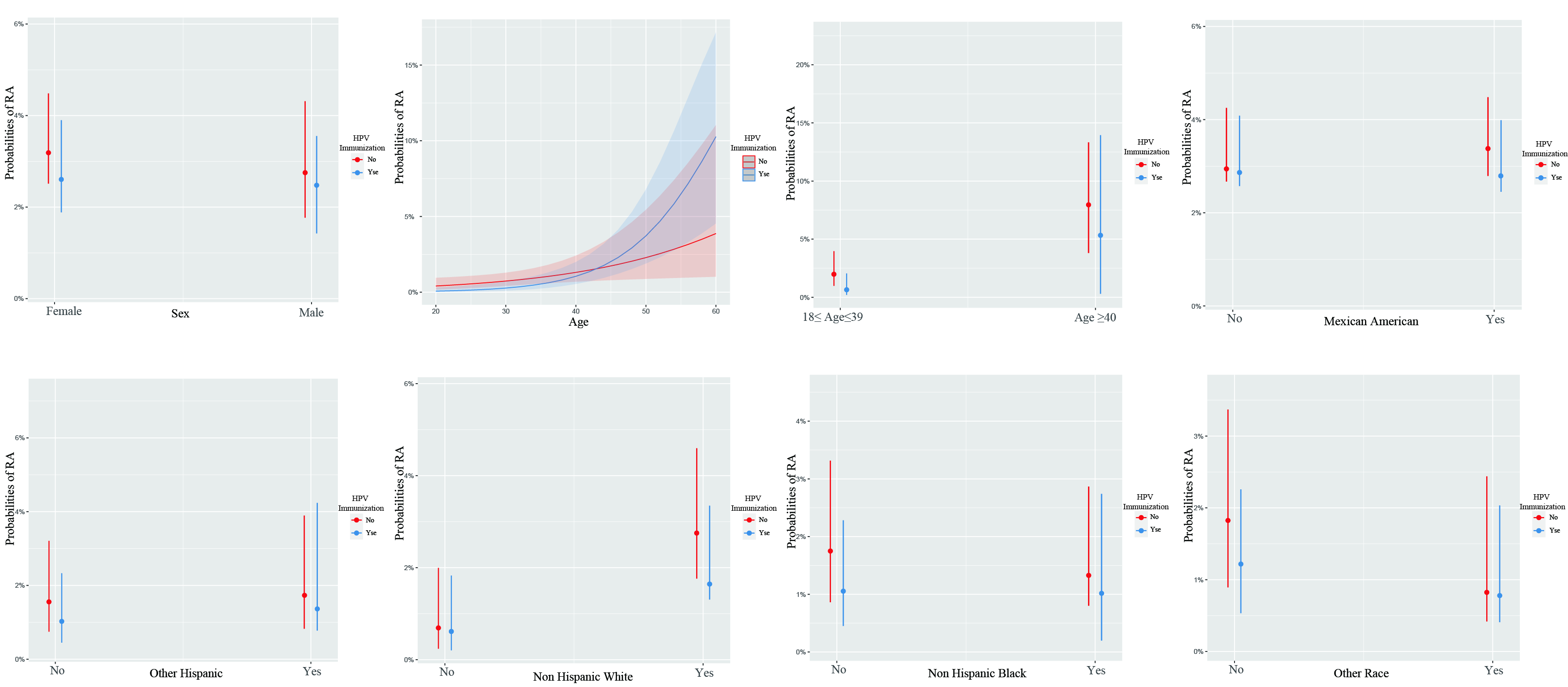


**Supplementary Figure 3** Subgroup analysis of model 2 in HPV immunization cohort. Model 2 was adjusted for the confounding covariates with the numerical missing ratio below 5%.

HPV, Human papillomavirus; RA, rheumatoid arthritis.

**Supplementary Table 1** Demographic characteristics of HPV infection cohort after weighting

|  | Before propensity score matched | | | After propensity score matched | | |
| --- | --- | --- | --- | --- | --- | --- |
|  | Infected | Uninfected | *P* value | Infected | Uninfected | *P* value |
|  | N = 47953290 | N = 82228157 |  | N = 43897184 | N = 43854959 |  |
| Age (years, mean ± SD) | 38.24 ± 11.53 | 38.91 ± 11.16 | **<0.001** | 38.16 ± 11.63 | 38.37 ± 11.37 | **<0.001** |
| Sex, n (%) |  |  |  |  |  |  |
| Male | 15878372 (33.1) | 39029229 (47.5) | **<0.001** | 15128383 (34.5) | 15416378 (35.2) | **<0.001** |
| Female | 32074918 (66.9) | 43198929 (52.5) | **<0.001** | 28768801 (64.5) | 28438581 (64.8) | **<0.001** |
| Race/ethnicity, n (%) |  |  |  |  |  |  |
| Mexican American | 7216148 (15) | 14303206 (17.4) | **<0.001** | 6860130 (15.6) | 7121405 (16.2) | **<0.001** |
| Other Hispanic | 5929230 (12.4) | 8361347 (10.2) | **<0.001** | 5334100 (12.2) | 5465832 (12.5) | **<0.001** |
| Non-Hispanic White | 16426234 (34.3) | 31916293 (38.8) | **<0.001** | 15451820 (35.2) | 15676294 (35.7) | **<0.001** |
| Non-Hispanic Black | 13798190 (28.8) | 14036832 (17.1) | **<0.001** | 11708696 (26.7) | 11012998 (25.1) | **<0.001** |
| Other race | 4583488 (9.6) | 13610479 (16.6) | **<0.001** | 4542438 (10.3) | 4578429 (10.4) | **<0.001** |
| Education, n (%) |  |  |  |  |  |  |
| Under high school | 37521137 (21.8) | 17325876 (21.1) | **<0.001** | 9653207 (22) | 9990512 (22.8) | **<0.001** |
| High school or equivalent | 11737418 (24.5) | 16440095 (20) | **<0.001** | 10436409 (23.8) | 10380520 (23.7) | **<0.001** |
| Above high school | 25783719 (53.8) | 48462187 (58.9) | **<0.001** | 23807569 (54.2) | 23483926 (53.5) | **<0.001** |
| Marital status, n (%) |  |  |  |  |  |  |
| Married/cohabiting | 23360958 (48.7) | 53818982 (65.5) | **<0.001** | 22355861 (50.9) | 23815128 (54.3) | **<0.001** |
| Widowed/divorced/separated | 10160345 (21.2) | 9413704 (11.4) | **<0.001** | 8578769 (19.5) | 7523875 (17.2) | **<0.001** |
| Never married | 14431987 (30.1) | 18995472 (23.1) | **<0.001** | 12962555 (29.5) | 43854959 (28.5) | **<0.001** |
| BMI (mean ± SD) | 29.74 ± 7.44 | 29.36 ± 7.39 | **<0.001** | 29.63 ± 7.44 | 29.77 ± 7.62 | **<0.001** |
| Smoking status, n (%) |  |  |  |  |  |  |
| Every day | 24765647 (51.6) | 34033356 (41.4) | **<0.001** | 22118261 (50.4) | 22235607 (50.7) | **<0.001** |
| Some days | 6114266 (12.8) | 9076000 (11) | **<0.001** | 5437210 (12.4) | 5296313 (12.1) | **<0.001** |
| Not at all | 17073376 (35.6) | 39118801 (47.6) | **<0.001** | 16341714 (37.2) | 16323038 (37.2) | **0.514** |
| Diabetes, n (%) |  |  |  |  |  |  |
| Yes | 4323814 (9) | 7332959 (8.9) | **<0.001** | 4017806 (9.2) | 3877614 (8.8) | **<0.001** |
| No | 42636713 (88.9) | 72966467 (88.7) | **<0.001** | 38917565 (88.7) | 39069350 (89.1) | **<0.001** |
| Borderline | 992763 (2.1) | 1928731 (2.3) | **<0.001** | 961814 (2.2) | 907995 (2.1) | **<0.001** |
| Hypertension, n (%) | 11688458 (24.4) | 17595971 (21.4) | **<0.001** | 10422561 (23.7) | 10486104 (23.9) | **<0.001** |
| Hyperlipidemia, n (%) | 11495437 (24) | 20950757 (25.5) | **<0.001** | 10637336 (24.9) | 10847103 (25) | **<0.001** |

The bold values means statistical significance

HPV, Human papillomavirus; BMI, body mass index; DM, diabetes mellitus.

**Supplementary Table 2** Demographic characteristics of HPV immunisation cohort after weighting

|  | Before propensity score matched | | | After propensity score matched | | |
| --- | --- | --- | --- | --- | --- | --- |
|  | Vaccinated | Unvaccinated | *P* value | Vaccinated | Unvaccinated | *P* value |
|  | N = 3694365 | N = 65294631 |  | N = 3520249 | N = 3791381 |  |
| Age (years, mean ± SD) | 28.39 ± 8.69 | 40.26 ± 10.93 | **<0.001** | 28.72 ± 8.77 | 28.46 ± 8.22 | **<0.001** |
| Sex, n (%) |  |  |  |  |  |  |
| Male | 1818448 (22.2) | 25187904 (38.6) | **<0.001** | 818448 (23.2) | 877952 (23.2) | **0.003** |
| Female | 2875917 (77.8) | 40106728 (61.4) | **<0.001** | 270181 (76.8) | 2913435 (76.8) | **0.003** |
| Race/ethnicity, n (%) |  |  |  |  |  |  |
| Mexican American | 410973 (11.1) | 11761354 (18) | **<0.001** | 410973 (11.7) | 469974 (12.2) | **<0.001** |
| Other Hispanic | 361961 (9.8) | 6342787 (9.7) | **<0.001** | 321705 (9.1) | 463451 (12.2) | **<0.001** |
| Non-Hispanic White | 1461570 (39.6) | 24342301 (37.3) | **<0.001** | 1410322 (40.2) | 1468910 (38.7) | **<0.001** |
| Non-Hispanic Black | 717337 (19.4) | 12132848 (18.6) | **<0.001** | 688309 (19.6) | 704189 (18.6) | **<0.001** |
| Other race | 742525 (20.1) | 10715343 (16.4) | **<0.001** | 688939 (19.6) | 693864 (18.3) | **<0.001** |
| Education, n (%) |  |  |  |  |  |  |
| Under high school | 409736 (11.1) | 13998977 (21.4) | **0.05** | 409736 (11.6) | 461708 12.2) | **<0.001** |
| High school or equivalent | 558187 (15.1) | 12897117 (19.8) | **0.043** | 551790 (15.7) | 589687 (15.6) | **<0.001** |
| Above high school | 2726443 (73.8) | 38398537 (58.8) | **<0.001** | 2558723 (72.7) | 2739992 (72.3) | **<0.001** |
| Marital status, n (%) |  |  |  |  |  |  |
| Married/cohabiting | 1681529 (45.5) | 43167756 (66.1) | **<0.001** | 1677128 (47.6) | 1542044 (40.7) | **<0.001** |
| Widowed/divorced/separated | 231782 (6.3) | 8616971 (13.2) | **<0.001** | 222007 (6.3) | 359096 (9.5) | **<0.001** |
| Never married | 1781054 (48.2) | 13509905 (20.7) | **<0.001** | 1621114 (46.1) | 1890248 (49.9) | **<0.001** |
| BMI (mean ± SD) | 28.89 ± 6.76 | 29.8 ± 6.62 | **<0.001** | 28.95 ± 6.8 | 28.91 ± 7.73 | **<0.001** |
| Smoking status, n (%) |  |  |  |  |  |  |
| Every day | 1803288 (48.8) | 29359912 (45) | 0.076 | 1700640 (48.3) | 1862363 (49.1) | **<0.001** |
| Some days | 497720 (13.5) | 7081471 (10.8) | 0.242 | 492830 (14) | 434830 (11.5) | **<0.001** |
| Not at all | 1393358 (37.7) | 28827784 (44.2) | **0.012** | 1326779 (37.7) | 1494195 (39.4) | **<0.001** |
| Diabetes, n (%) |  |  |  |  |  |  |
| Yes | 185648 (5) | 2873617 (4.4) | 0.328 | 161207 (4.6) | 165583 (4.4) | **<0.001** |
| No | 3481859 (94.2) | 61593526 (94.3) | 0.399 | 3332184 (94.7) | 3574575 (94.3) | **<0.001** |
| Borderline | 26858 (0.7) | 819306 (1.3) | 0.996 | 26858 (0.8) | 51230 (1.4) | **<0.001** |
| Hypertension, n (%) | 532930 (14.4) | 15408897 (23.6) | **<0.001** | 523155 (14.9) | 452732 (11.9) | **<0.001** |
| Hyperlipidemia, n (%) | 475019 (9.9) | 17140975 (26.3) | 0.187 | 472708 (13.4) | 361913 (9.5) | **<0.001** |

The bold values means statistical significance

HPV, Human papillomavirus; BMI, body mass index; DM, diabetes mellitus.
